# Changes of upright body posture in the sagittal plane of men and women occurring with aging – a cross sectional study

**DOI:** 10.1186/s12877-019-1096-0

**Published:** 2019-03-05

**Authors:** Huan Gong, Liang Sun, Ruiyue Yang, Jing Pang, Beidong Chen, Ruomei Qi, Xin Gu, Yaonan Zhang, Tie-mei Zhang

**Affiliations:** 1 The MOH Key laboratory of Geriatrics, Beijing Institute of Geriatrics, Beijing Hospital, National Center of Gerontology, No.1 Dahua Road, Dong Dan, Beijing, 100730 People’s Republic of China; 2 Department of Rehabilitation, Beijing Hospital, National Center of Gerontology, Beijing, 100730 People’s Republic of China; 3 Department of Orthopedics, Beijing Hospital, National Center of Gerontology, Beijing, 100730 People’s Republic of China

**Keywords:** Aging, Knee, Kyphosis, Lordosis, Neck, Photogrammetry, Posture, Thorax

## Abstract

**Background:**

Body posture is a fundamental indicator for assessing health and quality of life, especially for elderly people. Deciphering the changes in body posture occurring with age is a current topic in the field of geriatrics. The aims of this study were to assess the parameters of standing body posture in the global sagittal plane and to determine the dynamics of changes in standing body posture occurring with age and differences between men and women.

**Methods:**

The measurements were performed on 226 individuals between the ages of 20 to 89 with a new photogrammetry, via which we assessed five postural angles - neck, thorax, waist, hip and knee. The data were analyzed with t-test, one-way ANOVA, linear regression model and generalized additive model.

**Results:**

Among these segments studied here, neck changed most, while the middle segments of the body, waist and hip, were relative stable. Significant differences between men and women were found with respect to the angles of neck, thorax and hip. Three of the five postural angles were significantly influenced with aging, including increasing cervical lordosis, thoracic kyphosis and knee flexion, starting from no older than around 50 yrs. showed by fitting curve derived with generalized additive model. These changes were more marked among women. Besides, this study highlights the effects of age and gender on the complex interrelation between adjacent body segments in standing.

**Conclusions:**

The presented results showed changes in the parameters describing body posture throughout consecutive ages and emphasized that for an individualized functional analysis, it is essential to consider age-and gender-specific changes in the neck, thorax and knee. This paper presents useful externally generalizable information not only for clinical purposes but also to inform further research on larger numbers of subjects.

**Electronic supplementary material:**

The online version of this article (10.1186/s12877-019-1096-0) contains supplementary material, which is available to authorized users.

## Background

The increase of elderly people has been occurring at unprecedented rates. There will be 2 billion people aged 60 or over worldwide, accounting for 21% of the world’s population by 2050 [1, 2]. Along with these demographic changes, age - related diseases will bring a burden to society, for example, by an increase in demand for health services. However, the process of aging and the period of old age are still the least understood aspects of human development. Aging is defined as irreversible functional and structural changes in many organs and systems. These changes vary between individuals at different rates or extents. Deciphering the changes in body posture occurring with age is a current topic in the field of geriatrics which requires a great deal of study.

Body posture is a fundamental index for assessing health and quality of life, especially for elderly people [3, 4]. Such index can provide information for targeted health promotion [5], be used to assess healthy aging and for health abnormality alerts, such as osteoporosis [6], sarcopenia [7] and fall-risk estimation [8], because falling is the most frequent cause of unintentional injuries and often leads to death in elderly people [9]. As an aspect of body posture, maintaining proper sagittal alignment is an important determinant for proper spinal function [10], and sagittal alignment is becoming recognized as an important predictor of a patient’s outcome after spinal surgery [11]. Alterations in spinal alignment may serve as leading factor for diminished body biomechanics [10]. Stress concentration in unbalanced sagittal spine can lead to functional and structural pathology, such as pain and degeneration of disc and facet joints [12]. Recent studies support the idea that analysis of sagittal balance is a key point to optimize the management of spinal degenerative diseases [13–15].

In the normal population a standard sagittal balance does not exist. Generally, researchers take healthy young adults as normal control, and the body posture differences are found between younger and older people [16–19]. The sagittal alignment can be affected by aging related degeneration [19, 20]. Aging has a number of inevitable consequences regarding the axial skeleton [10], such as hypertrophic facet joints arthritis, degenerative disc disease, bone remodeling and atrophy of extensor muscles [10, 14]. These pathologies lead to the risk to progressively develop a global sagittal unbalance [21]. Spinal malalignment in elderly people greatly affects the quality of daily life [22]. Describing the specific changes of body posture with aging would allow for the development of targeted rehabilitation programs, such as exercise-base interventions, surgical interventions, spinal orthotics and postural taping, etc., starting from no matter before or after 60 years of age, and help for improving life quality of elderly people [18, 23].

Although aging is suggested to be associated with alterations in sagittal alignment, in turn body posture, changes of body posture occurring with aging is still a current issue in geriatrics needing further research. However, previous studies had some limitations as following: Firstly, most studies compared the body posture differences between populations only under 30 and over 60 years of age [16–19]. More investigations on changes occurring with age are required to get close to decipher the beginning of the age-related changes on body posture, which will be helpful for intervention of aging process. Secondly, most studies focused on only one segment, such as cervical [24], thoracic [25] and lumbar spine [10, 19]. As we have known, compensatory mechanisms contributing to keep the sagittal balance of the spine [14] involve in different segments, especially the adjacent ones, for instance, cervical and thoracic, thoracic and lumbar spine. Full-body assessment of sagittal plane is necessary to get global and thorough characteristics of sagittal axis changes.

The aims of this study were to assess the parameters of body posture in the global sagittal plane - from head to legs – in men and women from 20 to 89 years of age, and to determine the dynamics of changes in standing body posture in sagittal plane occurring with age and differences between men and women.

## Methods

### Subjects

The measurements were performed in 226 individuals between the ages of 20 to 89 yrs. The subjects were divided based on each decade of life. Seven groups were constructed according to ages, these being 20–29, 30–39, 40–49, 50–59, 60–69, 70–79 and 80–89 yrs. The composition of the groups is presented in Table 1. All subjects were ambulatory and able to remain in a standing position for tests. Height, body weight and body posture were measured. Ethical approval was gained from the Biomedical Ethics Committee of Beijing Hospital, Beijing, China. All subjects were informed about the purpose of the study, its protocol, and conditions for participation. We provided written statement of informed consent to participate in the study and for the examination results to be used for scientific purposes. Before testing, the participants completed a questionnaire collecting personal data and information on their current state of health.

**Table 1:** Number, age, height, body weight and BMI of each decade of life

Decade of life	Gender	Number	Age (yrs)	Height (cm)	Body weight (kg)	BMI (kg/m^2^)
20-29 yrs	M	11	24.8 ± 0.8	172.4 ± 1.0	72.0 ± 3.3	24.2 ± 1.1
	F	12	23.4 ± 0.8	162.8 ± 1.2 ***	57.0 ± 2.3 **	21.5 ± 0.7
30-39 yrs	M	8	34.1 ± 1.2	173.3 ± 3.0	70.5 ± 3.2	23.4 ± 0.7
	F	12	33.8 ± 1.0	162.5 ± 1.6**	55.2 ± 1.7*	20.9 ± 0.5
40-49 yrs	M	8	43.0 ± 1.1	172.3 ± 2.3	66.3 ± 4.8	22.2 ± 1.2
	F	8	46.1 ± 0.8	164.0 ± 1.3	59.8 ± 2.8	22.2 ± 1.0
50-59 yrs	M	9	53.2 ± 1.0	171.0 ± 1.9	68.4 ± 4.1	23.3 ± 1.0
	F	12	54.3 ± 0.9	159.3 ± 1.1 ***	64.3 ± 2.6	23.2 ± 0.6 #
60-69 yrs	M	23	66.7 ± 0.5	169.3 ± 1.0	68.0 ± 2.2	24.6 ± 0.4
	F	20	67.1 ± 0.5	158.1 ± 1.2 ***	58.2 ± 1.3 *	23.5 ± 0.6
70-79 yrs	M	53	73.6 ± 0.4	166.5 ± 0.8 $$	68.1 ± 1.3	23.9 ± 0.7
	F	26	74.0 ± 0.6	157.0 ± 0.9 ***##	58.1 ± 1.5 ***	23.7 ± 0.9
80-89 yrs	M	12	81.9 ± 0.6	165.8 ± 1.7	64.8 ± 2.1	25.4 ± 1.0
	F	12	81.8 ± 1.2	153.5 ± 2.0 ***##	55.9 ± 2.2	25.4 ± 1.0
ANOVA	M	124	–	P < 0.001	*P* = 0.734	*P* = 0.463
	F	102	–	P < 0.001	*P* = 0.073	*P* = 0.002

Data are expressed as Mean ± S.E.. M: male, F: female. * *p* < 0.05, ** *P* < 0.01, *** *P* < 0.001 vs male at the same decade of life. Height: $$ *p* < 0.01 vs 20-29 yrs. of the same gender, ## *p* < 0.01 vs 20–29, 30–39 and 40-49 yrs. of the same gender. BMI: # *P* < 0.05 vs 20–29 and 30–39 yrs.

### The photogrammetric method

#### Equipment, preparation and photographing procedure

**Fig. 1:**
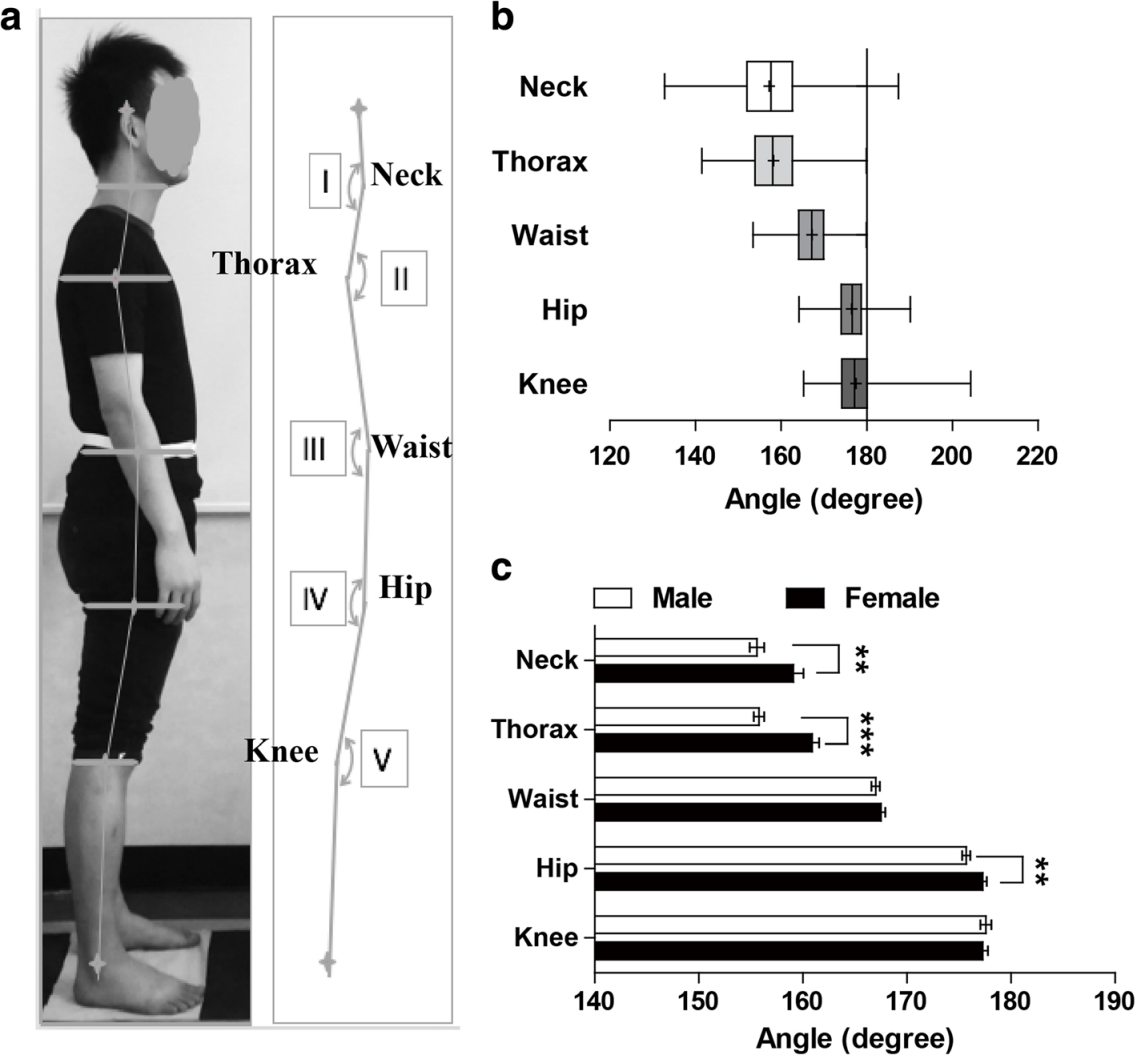
The angle definitions (**a**) and the overall body posture pattern (**b**) and gender differences (**c**). * *p* < 0.05, ** *P* < 0.01, *** *P* < 0.001

The photogrammetric method was used to evaluate body posture. In an attempt to minimize data collection error, all the subjects were measured and marked by two experienced researchers. They received comprehensive training in the use of study test protocols prior to commencement of the study. Strict protocols were used to ensure the correct labelling of markers, positioning of the subject and camera placement. All the subjects wore tightly-fit clothes with their ears, necks, knees and ankles exposed. A white belt was bound right up to iliac crest. The subjects were instructed to stand comfortably in a normal standing position with a relaxed head position, looking straight ahead as shown in Fig. 1. Photographs were obtained using a Nikon 1 J4 camera which was attached to a tripod and placed at a distance of 2.3 m from the subject. Spirit level adjustments placed on the top of the camera and the front of the lens to confirm horizontal and vertical alignments of the camera respectively. The tripod was secured in the correct position on the floor. Floor markers were used to standardize subject placement and to ensure that the subject’s right side was aligned perpendicular to the camera.

#### Digitizing and synthesizing posture data into angles

After sagittal plane photographs of usual, relaxed upright standing posture were completed, five horizontal straight lines were drawn on each photo with the midpoint of each line marked (Fig. 1a): the first line is right under the chin, the second line is through armpit, the third line is on the iliac crest which is marked by the bottom of the white belt, the fourth line is right through gluteal fold, and the fifth line is right through the poples. Besides these five midpoints, we labelled two other points on each photo: the highest point of ear marginal and lateral malleolus. Finally, we drew straight line between each two adjacent points and got five angles as following (Fig. 1a):

Angle I is the angle between head and neck and is named Angle Neck. Decreasing of this angle represents increasing cervical lordosis. Angle II is the angle between neck and trunk and is named Angle Thorax. Decreasing represents the accentuating thoracic kyphosis. Angle III is the angle between trunk and hip and is named Angle Waist. Decreasing represents the deepening lumbar lordosis and increasing represents the lumbar flattening. Angle IV is the angle between hip and thigh and is named Angle Hip. Angle V is the angle between thigh and shank and is named Angle Knee. Increasing represents the developing flexion of the knee in the sagittal plane. Angle values are reported in degrees. One researcher undertook all labelling and digitizing to eliminate inter-examiner error.

### Statistical analysis

Prior to statistical analysis, normal distribution for each numerical variable (of postural parameter) was examined. Values are expressed as the mean values with standard error. Statistical analysis was performed using SPSS software (version 17.0; SPSS; IL, USA). Independent sample t-test was used to estimate difference between genders. One-way ANOVA was used to estimate difference among ages or groups, followed by Bonferroni’s post hoc test. A two-tailed *P* < 0.05 was considered statistically significant. The correlation between angles was evaluated using Pearson’s coefficient of correlation and the linear regression model. GraphPad Prism 5 (GraphPad Software, USA) was used for graph plotting. The relationship between age and each angle was evaluated and plotted with generalized additive model (GAM) via R-package.

## Results

### The overall body posture characteristics of the population

We measured the height, body weight and calculated BMI of each subjects and showed the average value (mean ± standard error) of each group in Table 1. Although there are some significant differences on height and body weight between men and women at some decades of life, and in the same gender, there are some significant differences on height or BMI among different decades, however there has no significant association between height, body weight or BMI with any of the five angles we measured (Table 2,∣r∣ < 0.3). These results suggest that in the current population, the differences of the angles among different age groups are not from the differences of height, body weight or BMI.

**Table 2:** The correlation of height, weight and BMI with each body angle

Angle	Pearson’s correlation	Height (cm)	Weight (kg)	BMI (kg/m^2^)
Neck	r	−0.014	− 0.036	− 0.019
	P	0.842	0.596	0.778
Thorax	r	−0.123	− 0.130	− 0.081
	P	0.070	0.053	0.235
Waist	r	−0.054	− 0.236	− 0.258
	P	0.423	0.000	0.000
Hip	r	−0.112	0.070	0.171
	P	0.103	0.307	0.012
Knee	r	−0.053	0.017	0.073
	P	0.440	0.802	0.290

In the whole population of the current study, the average of each angle is shown in Table 3. The variation range of the angle is neck (132.8~187.4 degree) > knee (165.3~204.3 degree) > thorax (141.5~179.9 degree) > hip (164.2~191.3 degree) > waist (153.4~179.9 degree) (Fig. 1b). Besides, the coefficient of variation is as follows: neck (5.68%) > thorax (4.13%) > knee (3.07%) > waist (2.72%) > hip (2.35%). The data suggest that among these five segments in the current population, neck changes most, while the middle segments of the body, waist and hip are relative stable.

**Table 3:** Degrees of each angle at each decade of life

Decade of life	Neck (degree)	Thorax (degree)	Waist (degree)	Hip (degree)	Knee (degree)
20-29 yrs	162.8 ± 2.3 ***/##	163.3 ± 1.6 ***/###/$$$	167.5 ± 0.7	176.2 ± 0.9	175.4 ± 0.9
30-39 yrs	161.3 ± 2.0 */#	161.0 ± 1.2 $$	165.5 ± 1.0	176.1 ± 0.8	174.7 ± 1.0
40-49 yrs	161.7 ± 1.3 */#	162.4 ± 1.5 **/##/$$	167.1 ± 0.9	175.3 ± 0.8	172.6 ± 0.6
50-59 yrs	158.9 ± 1.8	161.0 ± 1.5 $$	166.6 ± 1.1	177.4 ± 0.9	177.9 ± 1.1
60-69 yrs	153.5 ± 0.9	156.2 ± 1.0	166.9 ± 0.6	176.9 ± 0.7	178.4 ± 0.9
70-79 yrs	154.6 ± 1.6	156.6 ± 0.6	167.7 ± 0.6	176.2 ± 0.5	178.4 ± 0.7
80-89 yrs	159.0 ± 0.6	154.6 ± 1.0	167.9 ± 1.0	176.8 ± 0.8	179.5 ± 0.8
total	157.2 ± 0.6	158.2 ± 0.4	167.2 ± 0.3	176.4 ± 0.3	177.5 ± 0.4
P	0.000	0.000	0.552	0.766	0.000
F	6.348	8.990	0.825	0.555	5.221

Data are expressed as Mean ± S.E. *, **, *** vs 60-69 yrs., #,##, ### vs 70-79 yrs., and $, $$, $$$ vs 80-89 yrs. *p* < 0.05, 0.01 and 0.001, respectively

Significant differences between men and women were found with respect to the angles of neck (*p* = 0.006), thorax (*p* < 0.001) and hip (*p* = 0.010) (Fig. 1c). Both the neck and thorax angles of men (155.7 ± 0.7 and 156.0 ± 0.5) are smaller than those of women (158.9 ± 1.0 and 160.8 ± 0.7). These differences mean deeper cervical lordosis and thoracic kyphosis of men than women. It suggests that men raise head higher and bend shoulders more forward than women. The smaller hip angle of men (175.8 ± 0.4) than women (177.2 ± 0.4) suggests that the pelvic shifts forward, tilts backward or hip joints extends more in men than in women.

### Changes of the body posture occurring with aging

#### Correlation between age and body posture

**Fig. 2:**
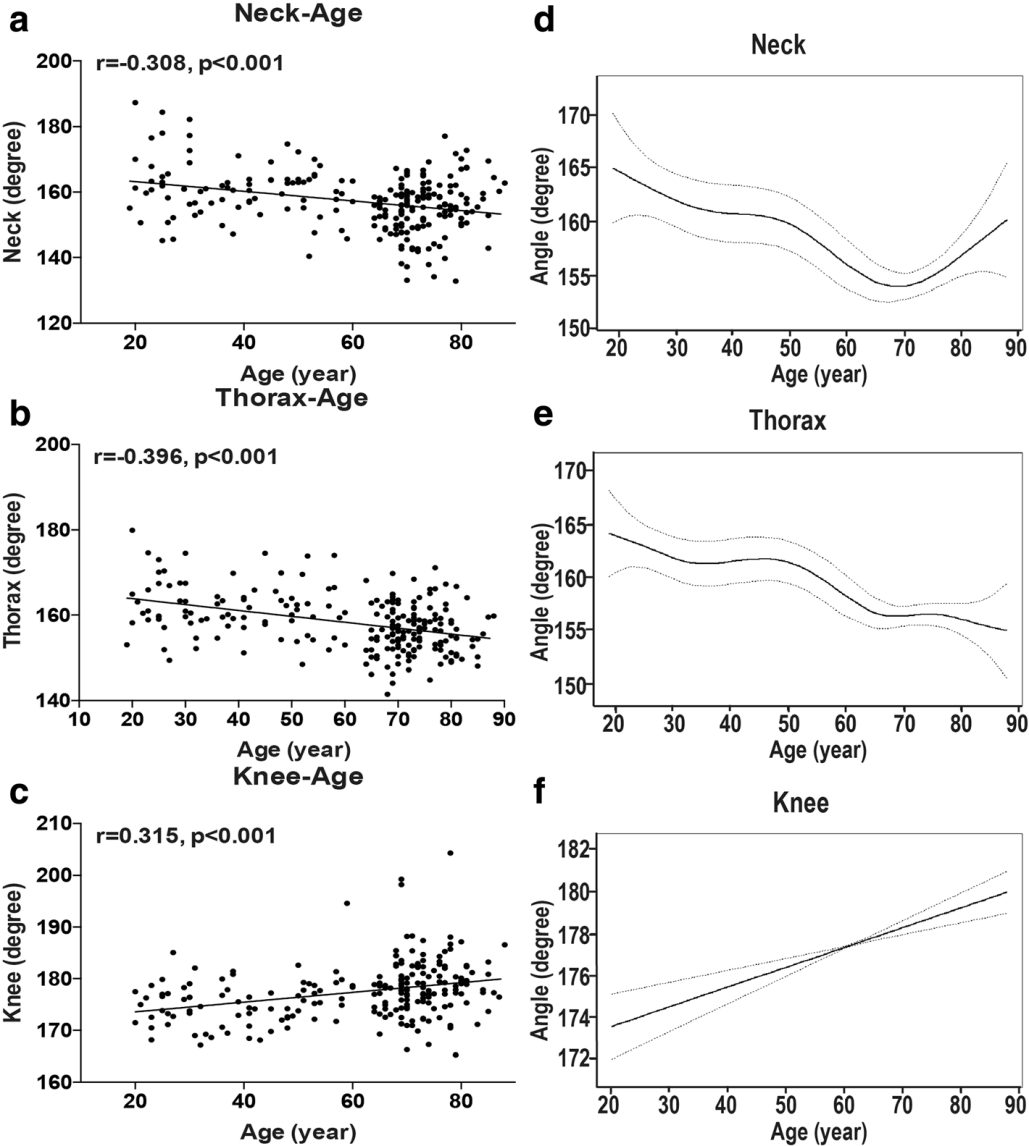
The correlation and curve-fitting between age and the angles of neck, thorax and knee. Neck (**a** and **d**), thorax (**b** and **e**) and knee (**c** and **f**). The curves were generated by generalized additive model. Dotted lines represent the 95% Confidence Interval

To decipher whether the body posture changes with aging, at first, we investigated the effects of age on the five angles through correlation analysis. Interestingly, correlations were found between age and angles of neck, thorax and knee (r > 0.3, *p* < 0.001, Fig. 2a-c), not between age and angle of waist or hip (r < 0.1, *p* > 0.1, Additional file 1: Figure S1). These results suggest that aging has effects on body posture, especially on segments of upper body and lower limbs. In consistence with the above analysis of correlation with age, there are significant differences among decades of life of the angles of neck, thorax and knee, but not of waist and hip (Table 3).

### The tendency of change of body posture with aging

To investigate further the dynamic changes of each angle with aging, we plotted fitting-curve with generalized additive model (GAM) (Fig. 2d-f). Before the age of 70 yrs., the overall trends of neck and thorax are similar: they are both decreasing with aging, especially from around the age of around 50 to 70 yrs., and they decrease little from around 30 to 50 yrs. (Fig. 2d and e). After the age of 70 yrs., the neck angle has a slight increasing trend, while thorax angle does not. These results suggest that both cervical lordosis and thoracic kyphosis develop with aging especially since around 50 yrs. and cervical lordosis recovers in population over around 70 yrs. On the contrary, the knee angle shows a steady increasing trend with aging (Fig. 2f), which suggests that when in standing position, the knees become more and more difficult to be extended out straightly and be flexed with aging.

### The influences of genders on the changes of body posture occurring with aging

**Fig. 3:**
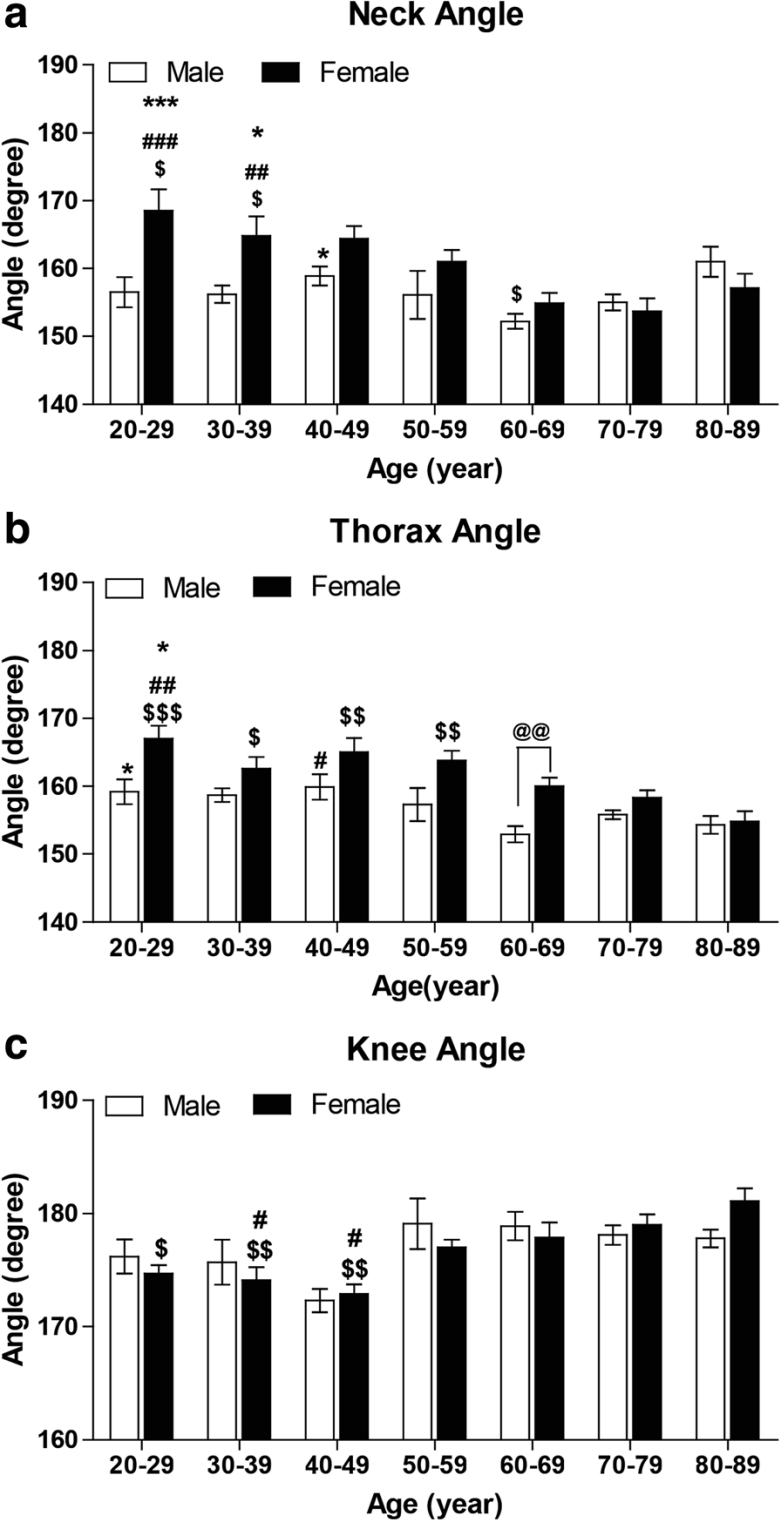
The gender effects on the changes of angles of neck, thorax and knee with aging. Neck (**a**), thorax (**b**) and knee (**c**). * p < 0.05 and *** *p* < 0.001 vs 60-69 yrs. of the same gender; # *p* < 0.05, ## *p* < 0.01 and ### p < 0.001 vs 70~70 yrs. of the same gender; $ p < 0.05 and $$ p < 0.01 vs 80-89 yrs. of the same gender; @@ p < 0.01

Since there are significant differences between men and women on the angles of neck and thorax, we analyzed further the influences of genders on the changes of body posture occurring with aging. Considering the changes throughout consecutive decades, we found that in women, there are significant differences of the angles of neck (F = 6.668, p < 0.001), thorax (F = 6.799, p < 0.001) and knee (F = 5.456, p < 0.001); while in men, only the thoracic angle has significant differences (F = 3.541, *p* = 0.003) (Fig. 3). The angles of waist (F = 0.327, *p* = 0.922; F = 1.135, *p* = 0.348), hip (F = 0.285, *p* = 0.943; F = 1.547, *p* = 0.172) in both men and women (Additional file 2: Figure S2) and the angle of neck (F = 2.108, *p* = 0.057) in men showed no significant differences.

For neck angle, a decreasing trend with aging is showed in women and no obvious trend has been observed in men, except that the angle of 80-89 yrs. is greater than that of 60-69 yrs. in men (Fig. 3a). These results suggest that, firstly, the cervical lordosis developing since 50 yrs. is mainly from women; secondly, the reducing of developed cervical lordosis after 70 yrs. is mainly because of men; thirdly, the gender difference of neck angle is mainly from subjects before 50 yrs. For thorax angle, women have a more identifiable decreasing trend than men throughout consecutive decades (Fig. 3b), which suggests that the thoracic kyphosis develops since 50 yrs. in both men and women, and thoracic kyphosis is deeper in men than women no matter before or after 50 yrs. For knee angle, there is an increasing trend with aging in women and a slight increase after 50 yrs. in men although without statistical significance (Fig. 3c). It suggests that maybe the knees start to be flexed in standing position in both men and women since about 50 yrs.

### The relationship between each two angles

**Fig. 4:**
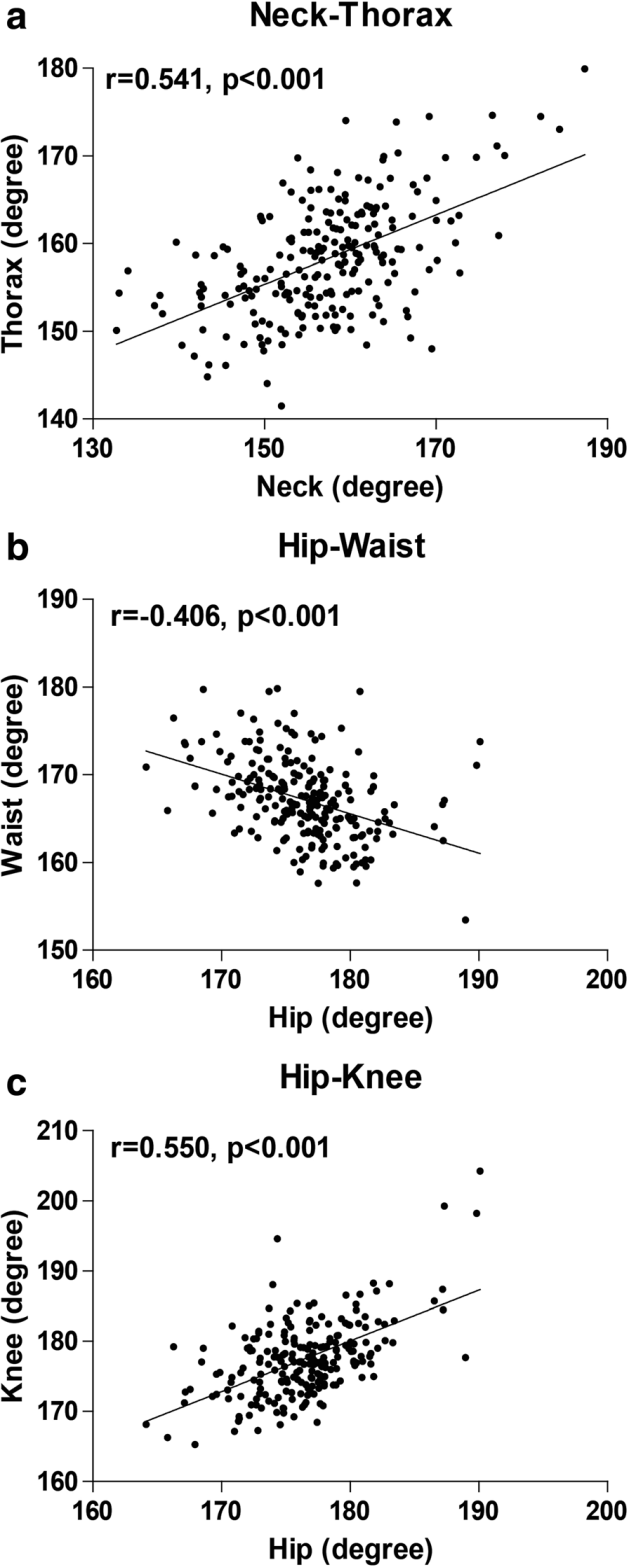
The correlation between the adjacent two angles. **a**. Neck and thorax. **b**. Hip and waist. **c**. Hip and knee

It has been reported that compensatory mechanisms contribute to keep the sagittal balance of the spine [14, 26]. For example, the loss of lumbar lordosis results in the loss of proper spinal alignment [27]. However, these consequences extend beyond the spinal column, as a cascade of compensatory mechanisms is involved to counteract spinal sagittal malalignment [28]. Mechanisms, including thoracic hypokyphosis, hip extension (pelvic retroversion around the hip joint), and increased flexion of the knee and ankle and so on, are commonly recruited [29–31]. To determine if our measurement of these angles can also reflect the compensatory mechanisms, we explored the relationship between each two angles through correlation analysis (Fig. 4). There indeed have moderate correlations between adjacent segments, including neck-thorax (Fig. 4a), waist-hip (Fig. 4b) and hip-knee (Fig. 4c), except that there has only little correlation between thorax and waist (r = 0.153, *p* = 0.021).

## Discussion

In the present study, we explored age and gender differences in relaxed standing body posture in the sagittal plane, with posture expressed as five angles derived from neck, thorax, waist, hip and knee, via a new photogrammetry. The results show significant changes with age in most parameters of body posture, including increasing cervical lordosis, thoracic kyphosis and knee flexion, especially starting from as early as around 50 yrs., and these changes were more marked among women than men. We believe that this paper present useful externally generalizable information not only for clinical purposes but also to inform further research on larger numbers of subjects, particularly to test angles of neck, thorax and knee.

### Effect of aging on body posture

There have been little data regarding the normal physiological sagittal changes with aging for the cervical and thoracic alignment before the current study. Subjects enrolled in the previous aging-related cervical alignment studies were usually before 80 yrs. [16, 24, 32] or were divided into old group after 60 yrs. [16, 33]. The previous aging-related thoracic alignment studies are in similar situation, since the researchers compared elderly people after 60 yrs. with young subjects aged 20-25 yrs., or only investigated elderly people [17, 18, 34]. In our study, we assessed the posture parameters in population covered from young to old age sequentially. Although Fon et al. measured thoracic kyphosis in subjects aged 2-77 yrs. [25], they reported only the mean degree of each decade of life and analyzed the correlation on age as linear regression. However, usually, the naturally dynamic changes with aging are not simply in linear. We applied GAM and got fitting-curve which presented the changes with aging closer to the true nature of the population compared to linear regression. Through these fitting-curves, we found that the tendency and degree of changes had discrepancy at different age stages.

The neck angle in this study gives a measure of the cervical lordosis, which is a useful clinical indicator of mid and/or lower cervical spine dysfunction. In general, cervical lordosis observed in the neutral position increases with aging [16, 32, 33, 35, 36]. Our research is the first report about the starting period of cervical lordosis development. Furthermore, we found for the first time in the asymptomatic subjects, the degree of cervical lordosis reduced from about 70 yrs., particularly in men. Similar to this, in a study of cervical spondylotic myelopathy patients, the lordotic angle measured by Cobb method also decreased in men and increased little in women after the decade of 80′ compared to before 80′ [35]. However, in another study, the cervical lordosis developed gradually from the 6th to 10th decade [36]. The discrepancy maybe because in the latter study, they enrolled much more women than men, since the lordosis angle of women is greater than men.

The thorax angle in this study gives a measure of the thoracic kyphosis. A decrease in this angle is considered to result in a ‘poking chin’ posture and hunchback. In previous studies, the deepening of thoracic kyphosis with age is seen in both men and women [17, 18, 25, 34]. In our study, we also found the thoracic kyphosis developed, especially deteriorated from around 50 yrs. Fon et al. reported that the thoracic kyphosis increase appears to be more obvious after 40 yrs. by means of a modification of the Cobb technique [25]. This discrepancy is maybe because of the difference of the methods, population selection, etc. However, both of these studies indicated thoracic kyphosis developed far earlier than 60 yrs.

In previous studies about changes of knee during aging, researchers usually investigated parameters of gait or walking ability [37–39], or in patients with deformity or after surgery [31, 40]. There are little studies assessed the knee posture when standing still in normal aging population. In the current study, we reported the flexion tendency of knee with aging in standing position, especially in women. Knee flexion is considered to be the last compensatory mechanism in case of sagittal imbalance [31, 40].

In consistence with our study about the middle segments of the body, waist and hip, some studies also found no significant difference in lumbar lordosis between younger and older volunteers [19, 23, 41]. However, many other studies reported a flattening of lumbar lordosis with aging [10, 34, 42, 43]. The possible reasons of this discrepancy include: since the middle segments of the body are relative stable and change in a narrow range, the assessment needs more accurate and more sensitive methods, such as radiographs; second, different parts of lumbar spine have different change tendency. For example, Dreischarf et al. reported that the lower lumbar spine retains its lordosis and mobility, whereas the middle part flattens and becomes less mobile in the older (> 50 yrs) compared to the younger age cohort (20–29 yrs) [42]. It suggests that different part of lumbar should be investigated separately. In study by Lee et al., although there was no significant difference in lumbar lordosis between the younger and older groups, when they separately analyzed lumbar lordotic angle as upper and lower lumbar lordosis, the distribution was different [19]. These findings suggest that the lumbar sagittal profile in a standing position is not affected by aging, but components of the lumbar spine with distinct anatomical and biomechanical functions do appear to be affected by aging.

### Effect of genders on body posture

We found that, although overall, both cervical lordosis and thoracic kyphosis were more accentuated in men than in women. However, the differences between men and women mainly occurred in younger ages - before 60 yrs. for neck angle and before 70 yrs. for thorax angle. After this age, the angles of two genders are close, while before this age, the younger, the bigger is the difference between two genders. Thus, both previous studies and the present study demonstrated that women had a greater tendency of accentuation of both cervical lordosis [24, 32, 36] and thoracic kyphosis [17, 25] than men during aging. Therefore, women developed greater compensatory lordosis of the cervical spine with age. Since the age-related progression of sagittal malalignment relates to some adverse effects [17, 44–46] and affects the quality of life [3, 4], the problem induced by deteriorating body posture relates to women significantly more often [47, 48]. Furthermore, the present study suggests that men and women have different normal body posture parameters and it is important to take the change tendency into account.

### Clinical implications

Considering the tight relationship between sagittal plane deterioration and quality of life measures [3, 4], there is growing interest of analyzing the sagittal plane and the non-deformity patients. Following a mild positive sagittal malalignment, the patient often begins recruiting mechanisms to compensate. Compensatory mechanisms are the patient’s progressive response to sagittal plane deterioration. Generally, elderly people exhibit greater thoracic kyphosis. The consequence of an accentuated thoracic curvature is mirrored in the cervical region with compensatory adjustments to head posture required to preserve forward gaze [14, 49]. In our study, we did find that greater accentuation of both cervical lordosis and thoracic kyphosis occurs with aging. Moreover, correlation between neck and thorax angles has been found and neck and thorax have similar change tendency with aging. Age-related progression of thoracic kyphosis may affect the quality of life in elderly people most profoundly. In osteoporosis patients, the lower quality of life, including physical and emotional roles, such as bodily pain and general health, are associated with increased thoracic kyphosis [50]. Extend adjacent segments of the kyphotic spine allowing for compensation of the sagittal unbalance but potentially inducing adverse effects [14, 17, 45]. Furthermore, considering the gender differences, the problem induced by deteriorating cervical lordosis and thoracic kyphosis relates to women significantly more often [47, 48]. On the other hand, assessment of the lower limbs is part of a full body sagittal plane analysis, including knee flexion angle. Knee flexion is another well-known compensatory mechanism for patients with severe degenerative spine and has already been widely reported [14, 51]. This report confirms the close interaction between spine and lower extremities.

For the aging population, comprehensive geriatric assessment (CGA) provides substantial insight into the comprehensive management of elderly people [52]. Developing concise and effective assessment instruments is helpful to carry out CGA widely to create a higher clinical value. CGA concerns the general health of elderly people and multidimensional and comprehensive scientific assessment of health status [52]. Physical health is one of the main domains of CGA. Since body posture is a fundamental index for assessing health and quality of life, it has potential to be involved in CGA. This can be applied to the elderly people in the community and in a variety of care settings.

### The advantages of this sagittal posture photogrammetry

Due to its non-invasive, reproducible, and sensitive testing characteristics, we decided to use the photogrammetry, which has been widely using in observational studies for body postural evaluation. Its use undoubtedly contributes to reducing exposure to radiation and thus enables the monitoring of postural treatment [18, 53]. Specifically regarding the application of photogrammetry in spinal evaluation, many studies have performed procedures to validate the technique [53–56]. However, the application of photogrammetry in postural evaluation is directly dependent on both the collection procedures and the mathematical methods used to provide measurements and postural diagnoses. In the current study, the five posture angles are considered useful and easily attained postural outcome measures. Besides the above advantages, our photogrammetry is simple and needs no special software. So it is appropriate for application to clinical studies, self-evaluation, intervention monitoring, population epidemiology survey etc.

### Limitations

As with any study, this investigation has some limitations. Firstly, the study sample was small. However, we endeavored to minimize distortion and generalization of the data by using various statistical tests. Further testing with larger subject numbers is required to be certain of these findings. Secondly, all the subjects are from Han Chinese and majority of them are mental workers. However, the height, body weight and BMI of our sample did not have significant influence on these body posture parameters, and these data are useful to help people of other background, who would prepare for an aged society, to understand age-related change. Thirdly, we evaluated body posture only in sagittal plane, not in coronal plane. Fourthly, as discussed above, we didn’t find significant changes of waist and hip, alterations of which have been reported during aging.

## Conclusions

The presented results showed changes in the parameters describing body posture throughout consecutive ages and emphasized that for an individualized functional analysis, it is essential to consider age-and gender-specific changes in the neck, thorax and knee, including increasing cervical lordosis, thoracic kyphosis and knee flexion, especially starting from as early as around 50 yrs., and these changes were more marked among women than men. This paper presents useful externally generalizable information not only for clinical purposes but also to inform further research on larger numbers of subjects.

## Additional files

Additional file 1: **Figure S1.** The correlation and curve-fitting between age and the angles of waist and hip. Waist (a and c) and hip (b and d).The curves were generated by generalized additive model (GAM). Dotted lines represent the 95% Confidence Interval. (JPG 1720 kb)

Additional file 2: **Figure S2.** Gender effects on the changes of angles of waist (a) and hip (b) with aging. (JPG 1519 kb)

## Abbreviations

GAM: Generalized additive model
yrs.: Years
