# Supplementary figures and images for: Changes of upright body posture in the sagittal plane of men and women occurring with aging – a cross sectional study
Source: BMC Geriatr. 2019 Mar 5;19:71. doi: 10.1186/s12877-019-1096-0 (PMC6402106; doi:10.1186/s12877-019-1096-0)

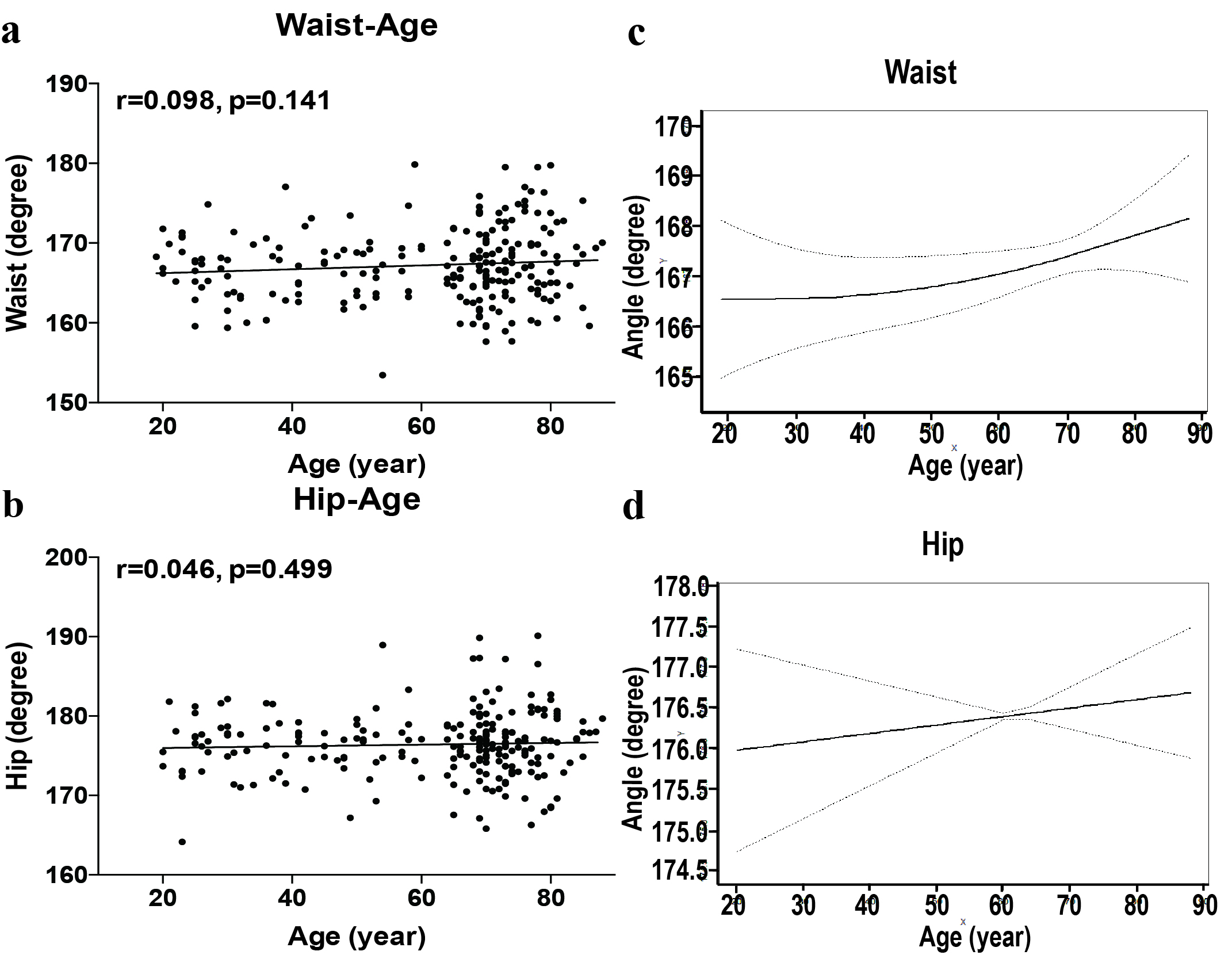

Supplement: Supplementary file 1 — Figure S1. The correlation and curve-fitting between age and the angles of waist and hip. Waist (a and c) and hip (b and d).The curves were generated by generalized additive model (GAM). Dotted lines represent the 95% Confidence Interval. (JPG 1720 kb) [file 12877_2019_1096_MOESM1_ESM.jpg]

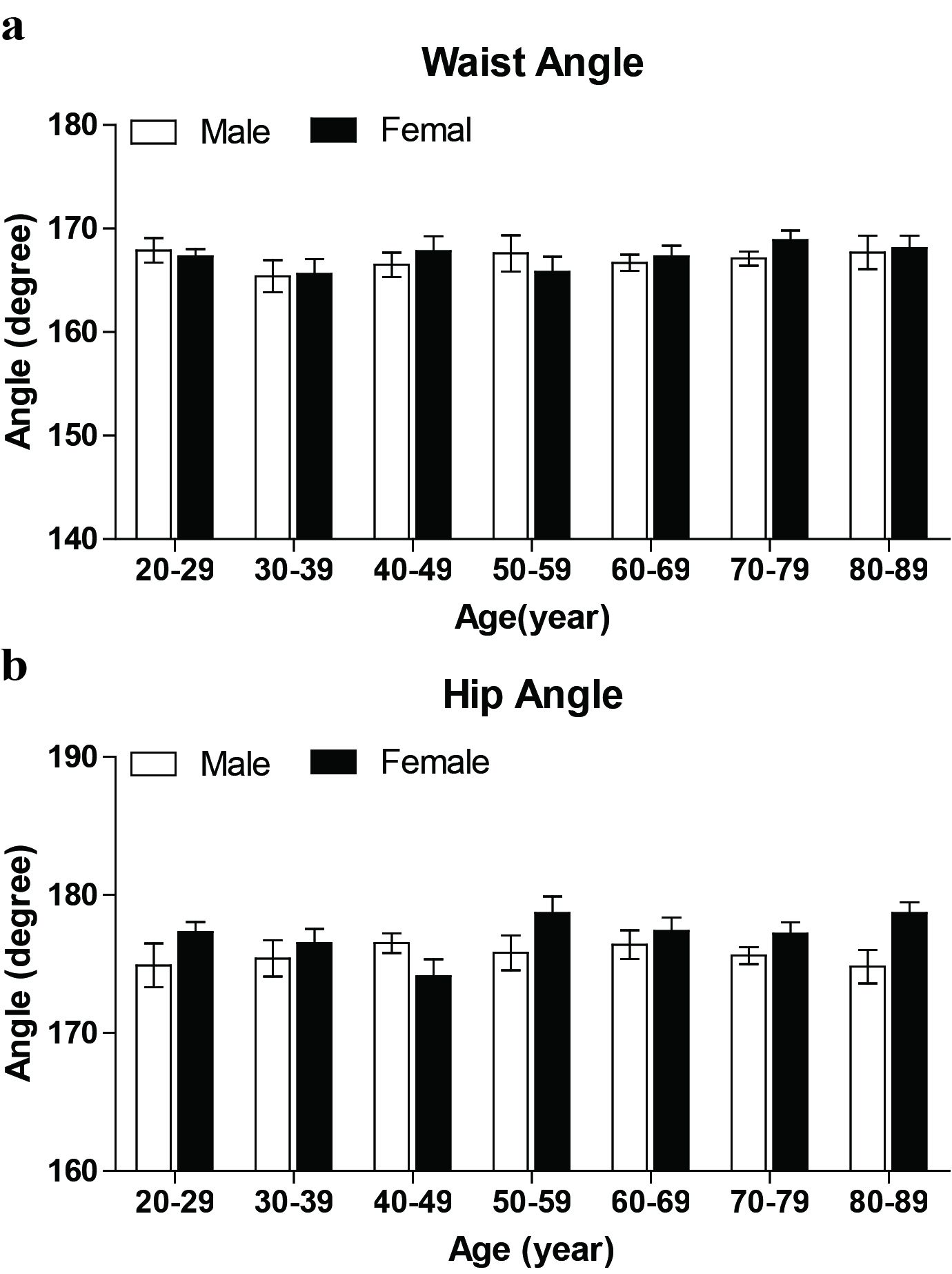

Supplement: Supplementary file 2 — Figure S2. Gender effects on the changes of angles of waist (a) and hip (b) with aging. (JPG 1519 kb) [file 12877_2019_1096_MOESM2_ESM.jpg]
